# Inhibition of human gastric cancer growth by cytokine-induced killer cells plus chemotherapy with or without cadonilimab in a mouse xenograft tumor model

**DOI:** 10.3389/fimmu.2025.1609320

**Published:** 2025-06-05

**Authors:** Wenwei Yang, Jingwei Liu, Tiantian Hou, Xu Lu, Lin Yang

**Affiliations:** ^1^ Department of Medical Oncology, National Cancer Center/National Clinical Research Center for Cancer/Cancer Hospital, Chinese Academy of Medical Sciences and Peking Union Medical College, Beijing, China; ^2^ Department of Oncology, Karh Biohealthcare Biotechnology (Zhejiang) Co., Ltd., Jiaxing, China; ^3^ National Center for Safety Evaluation of Drugs, Beijing Key Laboratory of Quality Control and Non-clinical Research and Evaluation for Cellular and Gene Therapy Medicinal Products, National Institutes for Food and Drug Control, Beijing, China

**Keywords:** gastric cancer, cytokine-induced killer cells, immune checkpoint inhibitors, chemotherapy, combination therapy, mouse xenograft model

## Abstract

**Background:**

Cytokine-induced killer (CIK) cell therapy has shown potent antitumor cytotoxicity. To date, no study has evaluated the efficacy and safety of combining CIK cell therapy with chemotherapy, with or without the immune checkpoint inhibitor (ICI) cadonilimab, for treating gastric cancer (GC).

**Methods:**

*In vitro* cytotoxicity, *in vivo* distribution, and acute toxicity of CIK cells were assessed. A nude mouse subcutaneous xenograft model of GC was established. To determine the antitumor effect of the CIK cells + chemotherapy regimen, 32 mice were randomized into the following four groups: control, CIK cells alone, chemotherapy alone, and CIK cells + chemotherapy. To evaluate the antitumor effect of CIK cells + chemotherapy supplemented with the cadonilimab regimen, mice subcutaneously inoculated with MGC803 cells were randomly assigned to the following eight experimental groups: vehicle, CIK cells, cadonilimab, chemotherapy, cadonilimab + chemotherapy, CIK cells + cadonilimab, CIK cells + chemotherapy, and CIK cells + cadonilimab + chemotherapy.

**Results:**

*In vitro* cytotoxicity assays indicated that CIK cells possessed good biocompatibility and sufficient therapeutic efficacy. An *in vivo* biodistribution assay revealed that CIK cells were mainly distributed in the spleen, lung, and liver. Acute toxicity analysis suggested that CIK cells had low toxicity. According to the tumor volume, the CIK cells + chemotherapy and chemotherapy-alone groups showed significantly higher tumor growth inhibition rates (34.2% and 50.8%, respectively) with well-tolerable toxicity than the control group (*p* < 0.01). The CIK cells + chemotherapy group exhibited a stronger, but not statistically significant, antitumor effect than the chemotherapy-alone group. In the safety and efficacy evaluation of CIK cells + chemotherapy + cadonilimab, the results showed that the tumor inhibitory effects of the cadonilimab + chemotherapy, CIK cells + chemotherapy, and CIK cells + cadonilimab + chemotherapy groups were significantly higher with tolerable toxicity than those of the CIK cells and cadonilimab groups (*p* < 0.05). The antitumor effect of the CIK cells + cadonilimab + chemotherapy regimen was also superior to that of the CIK cells + cadonilimab regimen (*p* = 0.0364). However, the tumor lysis ability showed no significant difference between the chemotherapy-based combination treatment groups and the chemotherapy-alone group.

**Conclusions:**

The combination of CIK cells and chemotherapy with or without ICIs can serve as a potential therapeutic option for treating GC, with promising efficacy and good tolerability.

## 1 Introduction

Gastric cancer (GC) ranks fifth in terms of morbidity and mortality rates among all cancer types worldwide, and it represents a severe public health threat (1). GC is a major health concern in East Asia, with the highest incidence rate in China (2). More than 80% of GC patients are in the advanced stage of the disease at the time of diagnosis. Chemotherapy remains the mainstream treatment approach for advanced GC. However, chemotherapy alone has limited efficacy in treating GC, with a median overall survival (OS) of 6–10 months (3).

Recently, based on the results of several clinical trials (e.g., CheckMate 649, KEYNOTE-859, ORIENT-16, and RATIONALE-305), anti-programmed cell death protein-1 (PD-1) plus chemotherapy has become the standard first-line treatment for HER2-negative, unresectable, locally advanced or metastatic gastric or gastroesophageal junction (G/GEJ) adenocarcinomas. However, the survival benefits were mostly observed in patients with high programmed death ligand 1 (PD-L1) expression. As reported previously, combining PD-1 and cytotoxic T lymphocyte antigen-4 (CTLA-4) inhibitors could enhance antitumor response in patients with multiple solid tumor types (4, 5). A phase III clinical trial (COMPASSION-15 study) showed that the combination of cadonilimab (anti-PD-1/CTLA-4 bispecific antibody) and chemotherapy substantially improved OS and progression-free survival (PFS) of GC patients even with low PD-L1 expression (combined positive score (CPS) < 5) (6).

Although the emergence of immune checkpoint inhibitors (ICIs) has offered survival benefits to patients with locally advanced or metastatic GC, these agents lack the required efficacy to meet the clinical demand (7, 8). Previous research has indicated that lymphocyte infiltration in tumor tissues positively correlates with the effectiveness of ICIs. Therefore, tumors with less lymphocyte infiltration may lead to poor efficacy of ICIs (9). Hence, novel therapeutic approaches are required to enhance antitumor immune responses.

Adoptive cell therapy (ACT) involves the collection of human autoimmune cells, proliferation of these cells through *in vitro* cultivation, enhancement of their targeted killing function, and re-inoculation of these cells into the patient’s body to kill cancer cells or mutated cells in blood and tissues (10). The current ACT approach utilizes tumor-infiltrating lymphocytes, lymphokine-activated killer (LAK) cells, dendritic cells (DCs), natural killer (NK) cells, cytokine-induced killer (CIK) cells, cytotoxic T lymphocytes, chimeric antigen receptor T cells, and T cell receptor-engineered T cells (11). ACT infusion is an alternative approach to increase intratumoral immune cell infiltration and augment the antitumor immune responses of the host (12).

CIK cells are a heterogeneous cell population obtained from human peripheral blood mononuclear cells (PBMCs) through *ex vivo* stimulation of multiple cytokines (13). The antitumor activity of CIK cells can be mainly attributed to the CD3^+^ CD56^+^ cells, which show an NK-like, major histocompatibility complex (MHC)-unrestricted antitumor activity without requiring prior antigen exposure or priming (14, 15). Moreover, *in vivo*, CIK cells can regulate and enhance the function of the host cellular immune response by secreting cytokines and chemokines. Therefore, CIK-based adoptive cellular immunotherapy might become a potential strategy for treating cancer. Previous research has demonstrated that CIK cells exhibit potent antitumor cytotoxicity against various tumor cells with tolerable side effects, both *in vitro* and *in vivo* (16–18). In recent years, several small-scale clinical studies have shown that CIK cell therapy combined with chemotherapy is a promising approach to improve the prognosis and quality of life of patients with locally advanced or metastatic GC (17, 19–25).

In the present study, we investigated the antitumor activity and toxicity of the combination of CIK cells, chemotherapy (oxaliplatin plus S-1), and cadonilimab in a nonclinical animal model of GC.

## 2 Methods

### 2.1 Cells and culture conditions

The human GC cell lines (MKN45 and MGC803) were obtained from the American Type Culture Collection (Manassas, VA, USA). These cell lines were cultured in Roswell Park Memorial Institute 1640 (RPMI 1640) medium supplemented with 10% fetal bovine serum (FBS, Excell Bio, Shanghai, China), streptomycin (100 µg/mL, Solarbio, Shanghai, China), and penicillin (100 U/mL, Solarbio) at 37°C under a 5% CO_2_ environment. The culture medium was replenished every 3 to 4 days. When the cell confluence reached approximately 80%, the cells were routinely passaged at a 1:3 ratio (approximately every 3 to 4 days). The cells in the logarithmic growth phase were utilized for subsequent experiments.

### 2.2 Establishment of the mouse subcutaneous xenograft model

Immunodeficient female B-NDG mice (Biocytogen Pharmaceuticals Co., Ltd., Beijing, China), female NCG mice (GemPharmatech Co., Ltd., Nanjing, China), and NPG mice (Beijing Vitalstar Biotechnology Co., Ltd., Beijing, China) were used for xenograft studies. All mice were reared under specific pathogen-free conditions by providing autoclaved food and water. Animal care and experiments were conducted according to the *Guide for the Care and Use of Laboratory Animals* of the National Institutes of Health. The animal use protocol for this study was reviewed and approved by the Laboratory Animal Ethical Committee, Chinese National Drug Safety Assessment and Monitoring Center (Approval Number: IACUC-2020-056).

In the experiment for determining the *in vivo* distribution of CIK cells, MKN45 cells (1 × 10^7^ cells/mouse) were subcutaneously injected into the right armpit of NPG mice on day 0. In the experiment for assessing the effect of the combination of CIK cells and chemotherapy, MKN45 cells (8.8 × 10^6^ cells/mouse) were subcutaneously inoculated into the back of B-NDG mice on day 0. To examine the effect of the combination of CIK cells, chemotherapy, and cadonilimab, MGC803 cells (5 × 10^6^ cells/mouse) were subcutaneously inoculated into the right flank of NCG mice on day 0.

### 2.3 CIK cell generation

PBMCs were obtained using freshly collected peripheral blood samples from healthy volunteers, who had provided signed informed consent for sample collection. PBMCs were isolated from the samples by Ficoll gradient density centrifugation. On day 0, PBMCs were seeded into a Corning T75 flask containing X-VIVO 15 media (Lonza, Switzerland) supplemented with recombinant human IFN-γ (R&D Systems, MN, USA) at 50 ng/mL concentration. After 24 h, 100 ng/mL of anti-CD3 pure human-functional grade antibodies (OKT-3, Miltenyi Biotec, CA, USA) and recombinant human interleukin-2 (Proleukin, Novartis, Switzerland) at 1,000 IU/mL concentration were added. From day 3, 50 ng/mL recombinant IL-15 (R&D Systems) was added to the medium at 2-day intervals. The cultures were maintained at 37°C under a 5% CO_2_ environment until injection into mice. On day 14, the cells were collected, washed with PBS solution, and centrifuged, and the supernatant was discarded. In this study, the tested CIK cell product was named TA cell.

### 2.4 Experimental design

#### 2.4.1 Experiment 1: *in vitro* cytotoxicity

The calcein-AM assay (26) was performed to determine the cytotoxicity of CIK cells *in vitro*. Human GC cells (AGS, HGC27, and MKN45), human non-small cell lung cancer (NSCLC) cells (HCC827), and human chronic myeloid leukemia cells (K562) were used as target cells. CIK cells were used as effector cells. Target cells were resuspended in RPMI-1640 medium at the final concentration of 1 × 10^6^ cells/mL and incubated with 1 μg/μL calcein-AM (Invitrogen Inc., city, country) at 37°C for 30 min. After two washes in RPMI 1640 medium, the cell density was adjusted to 2 × 10^5^ cells/mL. Effector cells and calcein-labeled target cells were then co-cultured in 96-well plates in triplicates for 4 h at 37°C with various effector-to-target cell (E: T) ratios, i.e., 50:1, 20:1, 10:1, 5:1, 2:1, 1:1, and 1:3. Wells with co-cultured target cells and PBS served as spontaneous release wells. Wells with co-cultured target cells and lysis buffer (5% Triton X-100) were considered maximal release wells. Following incubation, the plate was centrifuged for 5 min, and 100 μL of the supernatant was then transferred to another 96-well plate. The fluorescence intensity of the supernatant samples was measured with a microplate reader (Multiskan MK3, Thermo Fisher Scientific) with 490 nm excitation and 515 nm emission filters. Percent cytotoxicity of the assay was calculated as follows: Cytotoxicity (%) = [(experimental release - spontaneous release)/(maximal release - spontaneous release)] × 100%.

#### 2.4.2 Experiment 2: *in vivo* distribution

The *in vivo* distribution of CIK cells was determined by quantitative real-time PCR (qRT-PCR). A total of 126 NPG mice were assigned to three groups as follows (1): control group (21 males and 21 females) (2), nontumor-bearing mice group (21 males and 21 females), and (3) tumor-bearing mice group (21 males and 21 females). Tumor-bearing mice were generated by injecting MKN45 cells (1 × 10^7^ cells per mouse) subcutaneously into the right armpit of nude mice. Six days after tumor implantation, animals with a tumor volume of 100 mm^3^ were selected. The nontumor-bearing and tumor-bearing mice groups received an intravenous injection of CIK cells (4 × 10^7^ cells per mouse) through the tail vein. To determine the tissue distribution of CIK cells *in vivo*, we harvested various tissues (including blood, heart, liver, spleen, lung, kidneys, brain, testes, epididymides, uterus, ovaries, stomach, duodenum, colon, bone marrow, adipose tissue, and muscle) from the mice groups at different time points (3 h and 2, 7, 14, 28, 41, and 56 d post-dosing) and estimated the copy number of the human GAPDH (hGAPDH) gene by qRT-PCR, which reflected the injected CIK cell number.

#### 2.4.3 Experiment 3: acute toxicity

Forty-eight NPG mice were assigned to the following four groups, with 12 mice in each group (six males and six females): control group, low-dose group, mid-dose group, and high-dose group. High-dose, mid-dose, and low-dose groups were injected with 5 × 10^7^ cells/kg, 80 × 10^7^ cells/kg, and 250 × 10^7^ cells/kg CIK cells through the tail vein, respectively. The animals were observed twice daily (morning and evening) during the study to assess clinical symptoms. The body weight (BW) and food intake of mice in all groups were weighed weekly. The mice were monitored for 28 days and sacrificed for histopathological examination on the 29th day post-dosing.

#### 2.4.4 Experiment 4: efficacy of the combination of CIK cells and chemotherapy

To examine the antitumor effect of the CIK cells + chemotherapy regimen *in vivo*, 32 female B-NDG mice with subcutaneously inoculated MKN45 cells were divided into four groups (eight mice per group) and administered different treatment regimens. Mice in group 2 (G2) were dosed 1 day after tumor cell inoculation. Other mice were randomly assigned to group 1 (G1), group 3 (G3), and group 4 (G4) according to their tumor volume and weight on day 5 after inoculation. The dosing regimen of each group is shown in **Table 1**. The mice in the control group were administered an equal volume of the vehicle. The antitumor efficacy was determined based on BW, general status, tumor growth curve, and tumor weight (TW). The copy number of CIK cells in peripheral blood was assessed by quantitative PCR (qPCR). IFN-γ, TNF-α, IL-6, IL-4, and IL-10 levels in the tumor tissues were detected by ELISA. T-cell infiltration into tumor tissues was examined by immunohistochemistry (IHC). The levels of cytokines (IFN-γ, IL-2, TNF-α, IL-6, IL-4, and IL-10) in peripheral blood samples were detected by flow cytometry [fluorescence-activated cell sorting (FACS)].

**Table 1:** Treatment schedule involving CIK cells and chemotherapy.

Group	Drug		Dose	Application route	Day of administration
G1	Vehicle		NA	NA	NA
G2	CIK cells		5×10^7^ cells/mouse	*i.v.*	D1, D13, D28
G3	Chemotherapy	Oxaliplatin	4.2 mg/kg	*i.v.*	D5, D20
		S-1	6.9 mg/kg	*p.o.*	D5-11, D20-26
G4	CIK cells + Chemotherapy		CIK cells (5×10^7^ cells per mouse, i.v., D13, D28) Oxaliplatin (4.2 mg/kg, i.v., D5, D20) S-1 (6.9 mg/kg, p.o., D5-11, D20-26)		

NA, not available; i.v., intravenous; p.o., per os (oral administration).

#### 2.4.5 Experiment 5: efficacy of the combination of CIK cells, chemotherapy, and cadonilimab

**Figure 1:**
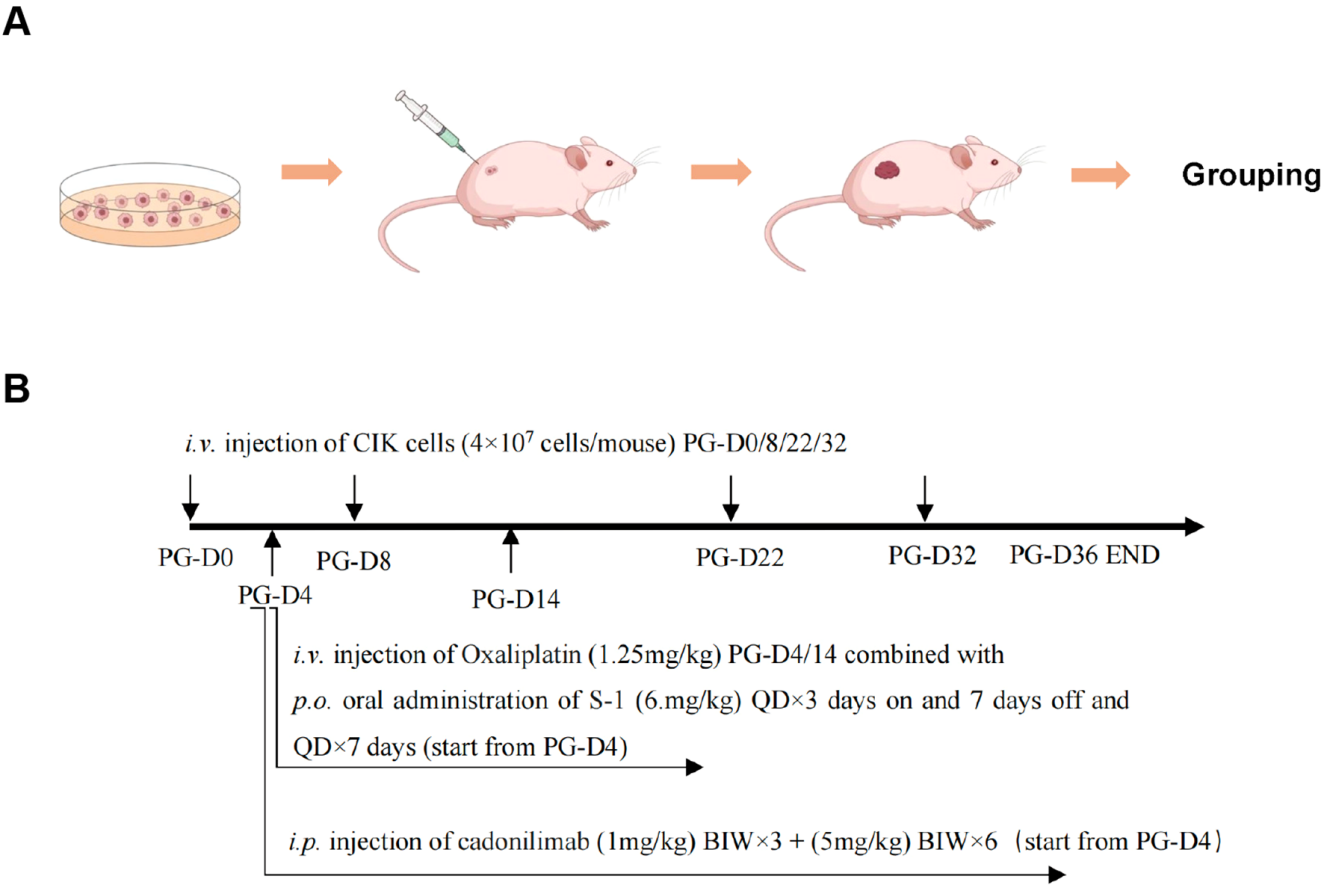
Schematic representation of the experimental design and the dosing procedure. **(A)** Diagram of establishing a mouse xenograft tumor model. **(B)** Schematic timeline for the drug administration. PG, post grouping; BIW, twice a week; QD, daily; p.o., per os (oral administration); i.v., intravenous; i.p., intraperitoneal.

To assess the antitumor effect of the combined regimen of CIK cells plus chemotherapy with cadonilimab, NCG mice with subcutaneously inoculated MGC803 cells were randomly assigned to eight experimental groups on day 5 (four or five mice per group). The schematic diagram of this experiment is provided in **Figure 1**, and complete details of drug administration doses and protocols are shown in **Table 2**. The mice in the control group were administered an equal volume of the vehicle. The antitumor efficacy was determined according to BW, general health status, tumor growth curve, and TW. The proportion of CIK cells was determined by FACS.

**Table 2:** The dosing regimen involving CIK cells, chemotherapy, and cadonilimab.

Group	N	Drug	Dose	Application route	Day of administration
G1	5	Vehicle	NA	*i.v.*	PG-D0/8/22/32
G2	5	CIK cells	4×10^7^ cells/mouse	*i.v.*	PG-D0/8/22/32
G3	4	cadonilimab	1 mg/kg	*i.p.*	BIW×3 (first three doses, started from PG-D4)
			5 mg/kg		BIW×6 (last six doses)
G4	5	Oxaliplatin	1.25 mg/kg	*i.v.*	PG-D4/14
		S-1	6.9 mg/kg	*p.o.*	QD×3 days on and 7 days off and QD×7 days (started from PG-D4)
G5	4	cadonilimab	1 mg/kg	*i.p.*	BIW×3 (first three doses, started from PG-D4)
			5 mg/kg		BIW×6 (last six doses)
		Oxaliplatin	1.25 mg/kg	*i.v.*	PG-D4/14
		S-1	6.9 mg/kg	*p.o.*	QD×3 days on and 7 days off and QD×7 days (started from PG-D4)
G6	5	CIK cells	4×10^7^ cells/mouse	*i.v.*	PG-D0/8/22/32
		cadonilimab	1 mg/kg	*i.p.*	BIW×3 (first three doses, started from PG-D4)
			5 mg/kg		BIW×6 (last six doses)
G7	5	CIK cells	4×10^7^ cells/mouse	*i.v.*	PG-D0/8/22/32
		Oxaliplatin	1.25 mg/kg	*i.v.*	PG-D4/14
		S-1	6.9 mg/kg	*p.o.*	QD×3 days on and 7 days off and QD×7 days (started from PG-D4)
G8	5	CIK cells	4×10^7^ cells/mouse	*i.v.*	PG-D0/8/22/32
		cadonilimab	1 mg/kg	*i.p.*	BIW×3 (first three doses, started from PG-D4)
			5 mg/kg		BIW×6 (last six doses)
		Oxaliplatin	1.25 mg/kg	*i.v.*	PG-D4/14
		S-1	6.9 mg/kg	*p.o.*	QD×3 days on and 7 days off and QD×7 days (started from PG-D4)

N, number of animals; PG, post grouping; BIW, twice a week; QD, daily; p.o., per os (oral administration); i.v., intravenous; i.p.: intraperitoneal.

### 2.5 Efficacy assessment

The tumor volume was measured using a vernier caliper twice a week. The tumor volume (TV) was calculated as follows: TV = L × W^2^/2, where L and W represent the long diameter and short diameter of the tumor, respectively. The relative tumor volume (RTV) was calculated as RTV = Vt/V0, where V0 is the initial TV before administration (Day 0) and Vt is the TV at the time of measurement (Day t). The evaluation index of the antitumor effect was the relative tumor proliferation rate: T/C (%) = (T_RTV_/C_RTV_) × 100%) (T_RTV_: the average RTV of the treatment group, C_RTV_: the average RTV of the vehicle control group). Tumor growth inhibition (TGI) was defined as TGI_TV_ (%) = (1 - T/C) × 100%. The BW of mice was monitored every other day by using an electronic balance and was recorded before and after drug administration. The general health status (including activity, feeding, and bowel movements) and survival of mice were observed and recorded during the experiment. At the end of the experiments, the mice were euthanized. The tumor tissues were excised and photographed, and the TW was measured. The TW of each group was estimated to calculate the tumor growth inhibition rate (TGI_TW_): TGI_TW_% =(1 - T/C)× 100% (T/C = the average TW of the treatment group/the average TW of the vehicle control group).

### 2.6 Flow cytometry

Peripheral blood samples were analyzed by flow cytometry. In the experiment for assessing the efficacy of CIK cells + chemotherapy with cadonilimab *in vivo*, peripheral blood samples from mice in the G2 (CIK cells), G6 (CIK cells + cadonilimab), G7 (CIK cells + chemotherapy), and G8 (CIK cells + cadonilimab + chemotherapy) groups were collected on days 7, 14, 17, and 36 after administration. Human cells (hCD45^+^, hCD3^+^, hCD4^+^, hCD8^+^, and hCD56^+^) in mouse peripheral blood samples were quantified by FACS.

During flow cytometry analysis, debris and cell aggregates were first excluded from mouse peripheral blood samples based on FSC-SSC characteristics to gate the single-cell population. Within the single-cell population, a CD45 vs SSC scatter plot was generated to gate CD45+ cells. From the CD45+ population, a CD3 vs SSC scatter plot was created to identify CD3+ T cells. Among the CD3+ cells, a CD4 vs CD8 scatter plot was generated to distinguish CD4+ and CD8+ subsets. Additionally, a CD3 vs CD56 scatter plot was plotted from the CD45+ population to gate CD3-CD56+ NK cells.

### 2.7 IHC

At the end of *in vivo* studies, tumor tissue samples were dissected from mice. Tissue specimens were fixed with 4% buffered formaldehyde, and paraffin-embedded sections were subjected to IHC analysis. Immunohistochemical staining of the tumor samples was used to evaluate the persistence and infiltration of CIK cells.

### 2.8 ELISA

At the end of the experiment, the tumor tissues were resected and collected to quantify cytokine levels. The levels of IFN-γ, TNF-α, IL-4, IL-6, and IL-10 were measured using ELISA sets for human IFN-γ, TNF-α, IL-4, IL-6, and IL-10 (BD Biosciences, USA), respectively.

### 2.9 Statistical analysis

GraphPad Prism 9.0 software (GraphPad Software, San Diego, CA, USA) and SPSS 19.0 software (IBM Corporation, NY, USA) were used for statistical description and analysis. One-way or two-way analysis of variance (ANOVA) was utilized for comparison among groups. Standard deviation (SD), standard error of the mean (SEM), and *p*-values were calculated using Student’s t-test and ANOVA. A *p*-value of < 0.05 was considered statistically significant.

## 3 Results

### 3.1 Cytotoxic activity of CIK cells against different human tumor cells

**Figure 2:**
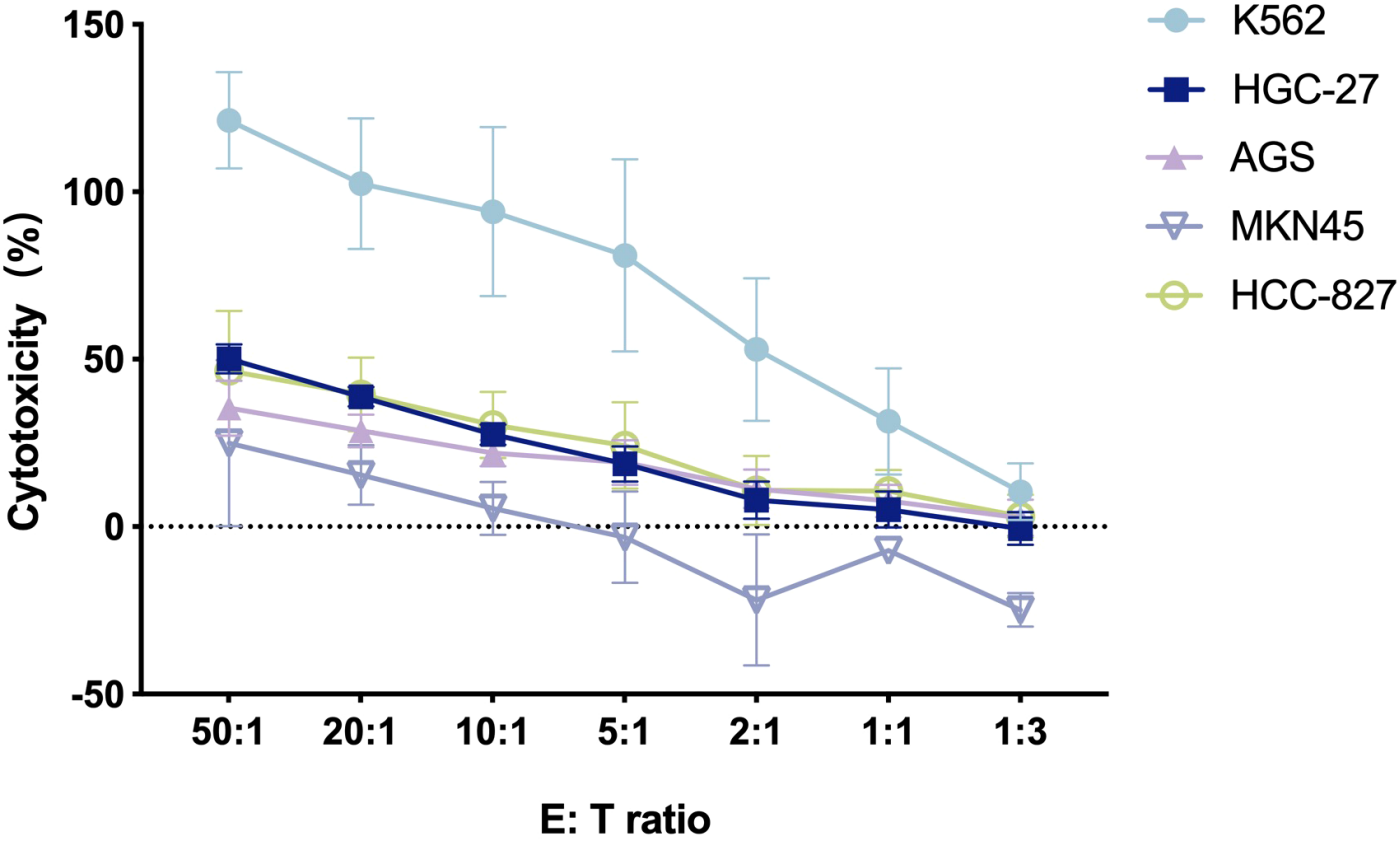
The cytotoxicity of CIK cells against various human tumor cells at different E/T ratios [n = 3, mean ± standard deviation (SD)].

The cytotoxicity of CIK cells was examined under *in vitro* conditions in a calcein-AM cytotoxicity assay. The results indicated that CIK cells showed specific cytotoxic activity against various human cancer cell lines, and the cytotoxicity level increased as the E: T ratio increased between 1:3 and 50:1 (**Figure 2**). K562 cells were most susceptible to CIK cell-mediated cytotoxicity with the highest tumor cell lysis rate (121.3% ± 14.4%) at the E: T ratio of 50:1. The highest cytotoxicity rates of CIK cells toward GC cells HGC-27, AGS, and MKN-45 were 50.1% ± 4.3%, 35.4% ± 8.2%, and 24.9% ± 24.8%, respectively. CIK cells also showed cytotoxic effects on the human NSCLC cells (HCC827), with the highest tumor cell lysis rate of 46.6% ± 17.9%. Overall, the results confirmed that CIK cells killed various human cancer cells *in vitro*. The antitumor effect of CIK cells was dose-dependent, which indicated that the strong cytotoxic effect was achieved through the non-MHC-restricted pathway.

### 3.2 Biodistribution of CIK cells *in vivo*

**Figure 3:**
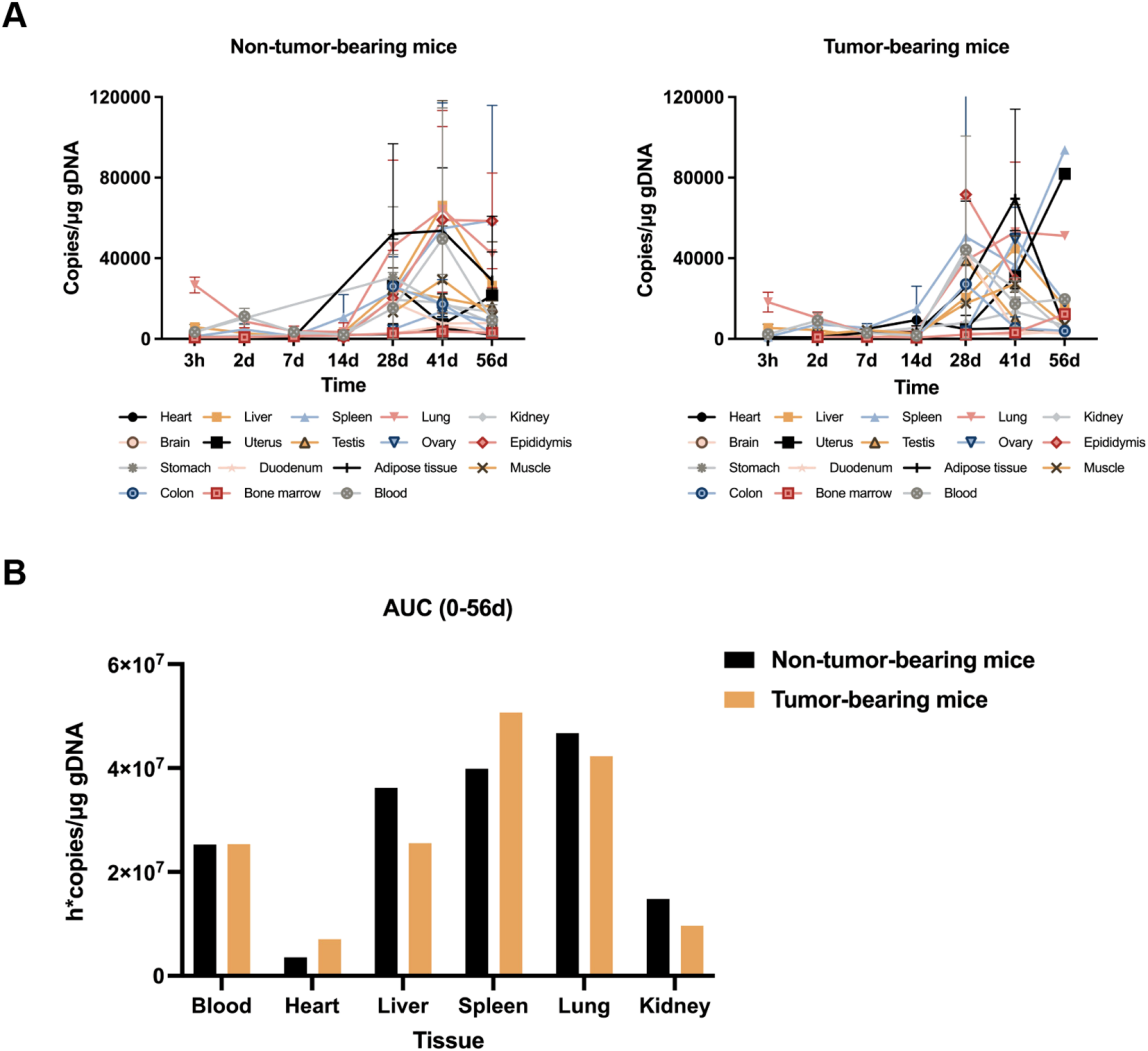
*In vivo* biodistribution of CIK cells. **(A)** Blood and various tissues hGAPDH gene copies concentration-time profiles after CIK cells administration to non-tumor-bearing mice and tumor-bearing mice (mean ± SD). **(B)** CIK cell accumulation in blood and various tissues from 0 to 56d post-dose.

The distribution of CIK cells *in vivo* in mice was assessed by qRT-PCR, wherein the abundance of the hGAPDH gene in various organs of mice was estimated at different time points (3 h and 2, 7, 14, 28, 41, and 56 d) after injecting CIK cells. Variations in the copy number of the hGAPDH gene were consistent between the nontumor-bearing mice group and the tumor-bearing mice group. As shown in **Figure 3A**, *hGAPDH* gene copies showed abundance in various tissues at different time points. In peripheral blood, the number of *hGAPDH* gene copies peaked at 2 d and then declined. At 28 days after drug administration, the copy number of the *hGAPDH* gene began to increase and reached a second peak at 41 d. In the lung, liver, heart, and kidney, the *hGAPDH* gene copy number exhibited an overall declining trend from 3 h to 14 d, then gradually increased, and peaked at 41 d. The spleen tissue is an important immune organ; *hGAPDH* gene copies were detected in the spleen tissue at 3 h and exhibited an overall upward trend from 14 d to 56 d. The *hGAPDH* gene copies in adipose tissue and bone marrow remained relatively low from 3 h to 14 d, started to increase at 14 d, and peaked at 41d. In the reproductive organs (uterus, ovary, testis, and epididymis), stomach, skeletal muscle, duodenum, and colon tissues, *hGAPDH* gene copies were mostly detected after 28 d. Overall, CIK cells were mainly distributed in the spleen, followed by the lung, liver, and peripheral blood, with a relatively low distribution in the kidney and heart tissues (**Figure 3B**). In most tissues, the gene copy number was significantly increased from 28 to 41 d, indicating the activation of CIK cells. Both male and female mice showed consistent trends in CIK cell distribution.

### 3.3 Acute toxicity of CIK cells

An acute toxicity study was performed to evaluate the potential toxicity of CIK cells. During clinical observation, female mice in the mid-dose and low-dose groups appeared normal, with no signs of poisoning and no death. In the high-dose group, 50% of the mice experienced symptoms such as piloerection and hunched posture. Compared to female mice, several male mice in all groups presented symptoms, including piloerection, epilation, and hunched posture, which might be associated with xenogeneic graft-versus-host disease (xGVHD). On the 26th day after administration, one female mouse in the high-dose group died. Microscopy examination revealed that this mouse had xGVHD-associated histopathological changes and showed severe bone marrow failure. Therefore, the cause of death may be related to the occurrence of severe xGVHD in this mouse. CIK cell administration showed no significant effect on mouse BW and food intake in the mid-dose and low-dose groups. In the third week, mice in the high-dose group had lower feed intake than control mice, and the increase in their BW was suppressed. The BW recovery of female mice began in the fourth week after administration. The results of the evaluation of acute toxicity are shown in **Table 3**.

**Table 3:** The body weight of mice during acute toxicity (mean ± SD, g).

Sex	Group	Day of grouping	Days after administration					
			0	7	14	21	28	29
Female	Control group	20.5 ± 2.0	20.2 ± 1.8	20.9 ± 1.9	21.5 ± 1.5	22.5 ± 1.3	23.1 ± 1.3	22.8 ± 1.1
	Low-dose group	20.2 ± 1.4	19.9 ± 1.6	21.0 ± 1.6	21.5 ± 1.8	22.0 ± 2.0	22.7 ± 1.7	23.0 ± 2.4
	Mid-dose group	20.1 ± 1.3	20.1 ± 1.5	20.5 ± 1.1	21.1 ± 1.1	22.2 ± 0.8	22.7 ± 1.5	22.2 ± 1.5
	High-dose group	20.0 ± 1.3	20.0 ± 1.3	20.2 ± 1.9	21.4 ± 1.9	20.2 ± 1.9	20.2 ± 2.4*	20.0 ± 2.5
Male	Control group	24.1 ± 1.8	24.1 ± 2.0	25.5 ± 2.4	27.7 ± 2.6	29.3 ± 2.5	30.5 ± 2.9	30.9 ± 2.8
	Low-dose group	24.0 ± 1.5	24.1 ± 1.5	25.1 ± 1.6	26.8 ± 1.4	28.2 ± 1.3	29.3 ± 1.9	29.2 ± 1.5
	Mid-dose group	23.9 ± 1.4	24.0 ± 1.5	24.7 ± 2.0	26.5 ± 1.9	27.6 ± 2.1	28.2 ± 2.0	27.8 ± 1.7
	High-dose group	23.8 ± 1.3	23.5 ± 0.9	24.6 ± 0.7	26.2 ± 1.0	23.7 ± 2.0**	21.9 ± 1.7**	21.7 ± 1.7

^*^compared with the vehicle control group, *p* < 0.05; ^**^compared with the vehicle control group*, p* < 0.01.

### 3.4 *In vivo* antitumor effect of the combination of CIK cells and chemotherapy

#### 3.4.1 Changes in BW

**Figure 4:**
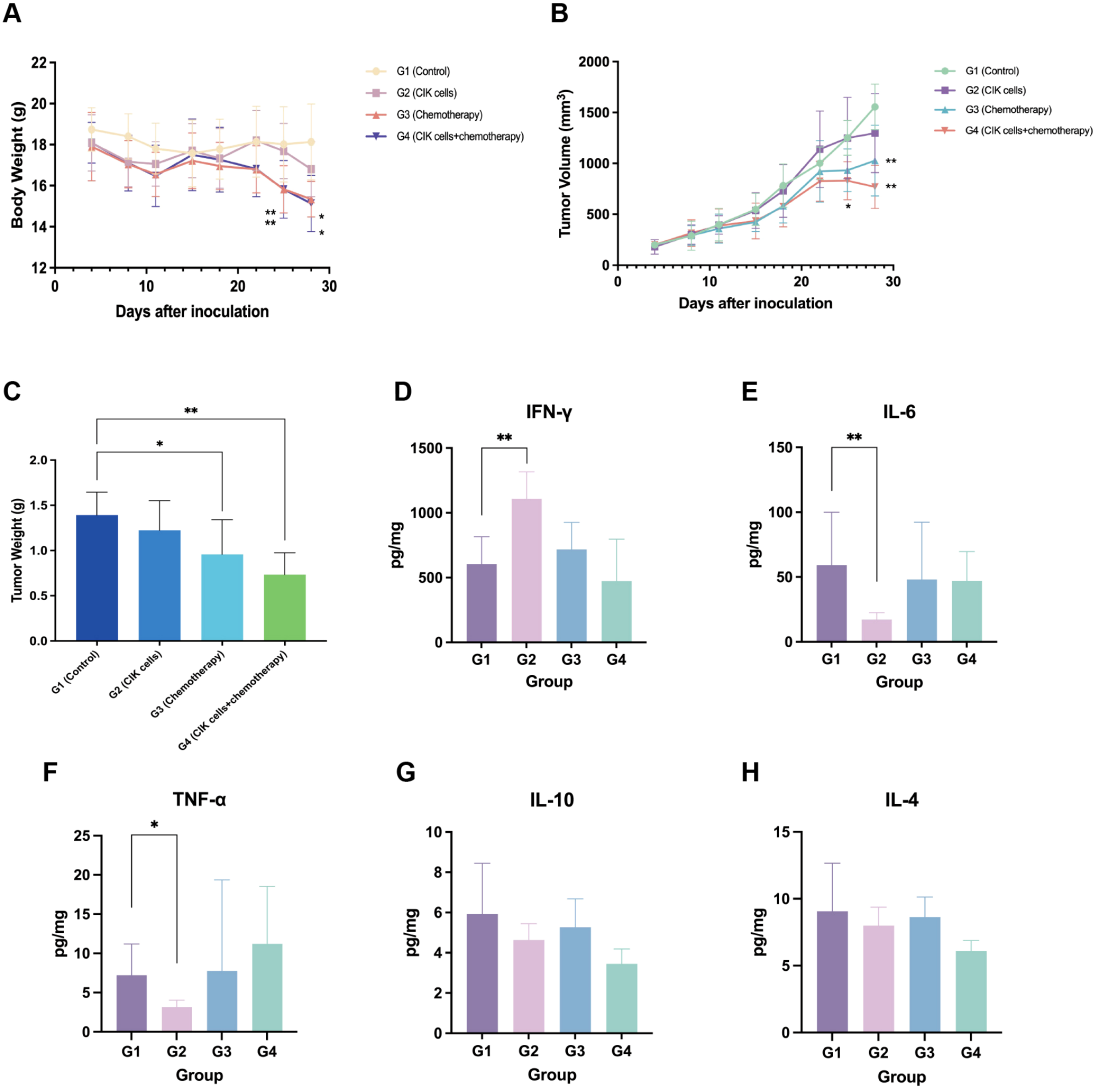
Antitumor effect of combination CIK cells with chemotherapy. **(A)** The body weight change curve of mice after administration. **(B)** Tumor progression after administration with CIK cells and/or chemotherapy. **(C)** Tumor weight of mice 29 days after administration. **(D–H)** Levels of IFN-γ, IL-6, TNF-α, IL-10, and IL-4 in tumor tissues. *, compared with the vehicle control group, *p* < 0.05; **, compared with the vehicle control group, *p* < 0.01; mean ± SD, n = 8.

The mouse subcutaneous xenograft model was used to evaluate the antitumor effects of the combination of CIK cells and chemotherapy. Following drug administration, all animals showed good general conditions with normal behavior, feeding, and drinking within 3 weeks; this finding suggested that the tested CIK cell-based regimen was safe and adequately tolerable. Twenty-two days after administration, the BW of mice in the G3 and G4 groups showed a significant decrease. At the end of the experiment, the animals in the G3 and G4 groups had lower BW (by 2.6 g and 3.0 g, respectively) than those before administration. The mean BW of the G2 group mice also decreased by 1.3 g at the end of the experiment. The mean BW of the G3 and G4 groups was significantly different from that of the control group (G1) at 25 and 28 d, respectively (p < 0.05). The decrease in the BW of mice in the G3 and G4 groups might be associated with the cytotoxicity of chemotherapeutic drugs and GVHD. The changes in BWs are shown in **Figure 4A**.

#### 3.4.2 Changes in tumor volume and TGI

On day 28, the average tumor volume of the control group (G1) was 1,556.2 ± 223.3 mm^3^, and those of the G2, G3, and G4 groups were 1,298.6 ± 387.6, 1,027.5 ± 347.0, and 770.5 ± 212.2 mm^3^, respectively. The TGI rates based on the tumor volume were 7.8%, 34.2%, and 50.8% for the G2, G3, and G4 groups, respectively. The TGI_TV_ rates of the combination group and the chemotherapy group were significantly higher than that of the control group (*p* < 0.01). However, CIK cells alone had a limited effect on the growth inhibition of gastric tumors. Moreover, the TGI_TV_ rate of the combination group was higher than that of the chemotherapy group, but not significant (*p* > 0.05). The change in the tumor volume for each group is shown in **Figure 4B**.

#### 3.4.3 TW inhibition rate

At the end of the experiment (29 d), the mice were euthanized, and the tumors were dissected, weighed, and photographed. The average TW in each group was calculated and compared (**Figure 4C**). The TWs in the G3 and G4 groups were significantly lower than that of the control group (*p* < 0.05). The inhibition rates of TW (TGI_TW_) in the G3 and G4 groups were 31.2% and 47.2%, respectively. The TGI_TW_ of the G3 and G4 groups was significantly higher than that of the control group; this finding showed that both CIK cells + chemotherapy regimen and chemotherapy alone could inhibit tumor growth. Although the result was not statistically significant, the CIK cells + chemotherapy combination group exhibited a trend toward a higher TGI_TW_ than the chemotherapy-alone group. The TGI_TW_ in the G2 group was only 12.1%, which suggested that CIK cells alone exhibited no apparent tumor suppressive effect.

#### 3.4.4 Infiltration of CIK cells in the tumor tissues of mice

The infiltration of T cells at the tumor site was evaluated by IHC. The results showed that T cell infiltration in the tumor tissues could be detected in the G2 and G4 groups after CIK cell administration. The tumor margin, but not the stroma or tumor core, was the primary site for the active infiltration of T cell subsets (**Supplementary Figure 1**).

#### 3.4.5 Cytokine secretion in tumor tissues

At the end of the experiment, ELISAs were conducted to detect the cytokines secreted in tumor tissues. The resected tumor mass from the G2 group showed an elevated IFN-γ level and reduced TNF-α and IL-6 levels; this finding suggested the infiltration of activated T cells into the tumor tissues (**Figures 4D–H**).

### 3.5 *In vivo* antitumor effect of the combination of CIK cells, chemotherapy, and cadonilimab

#### 3.5.1 Changes in BW of mice

**Figure 5:**
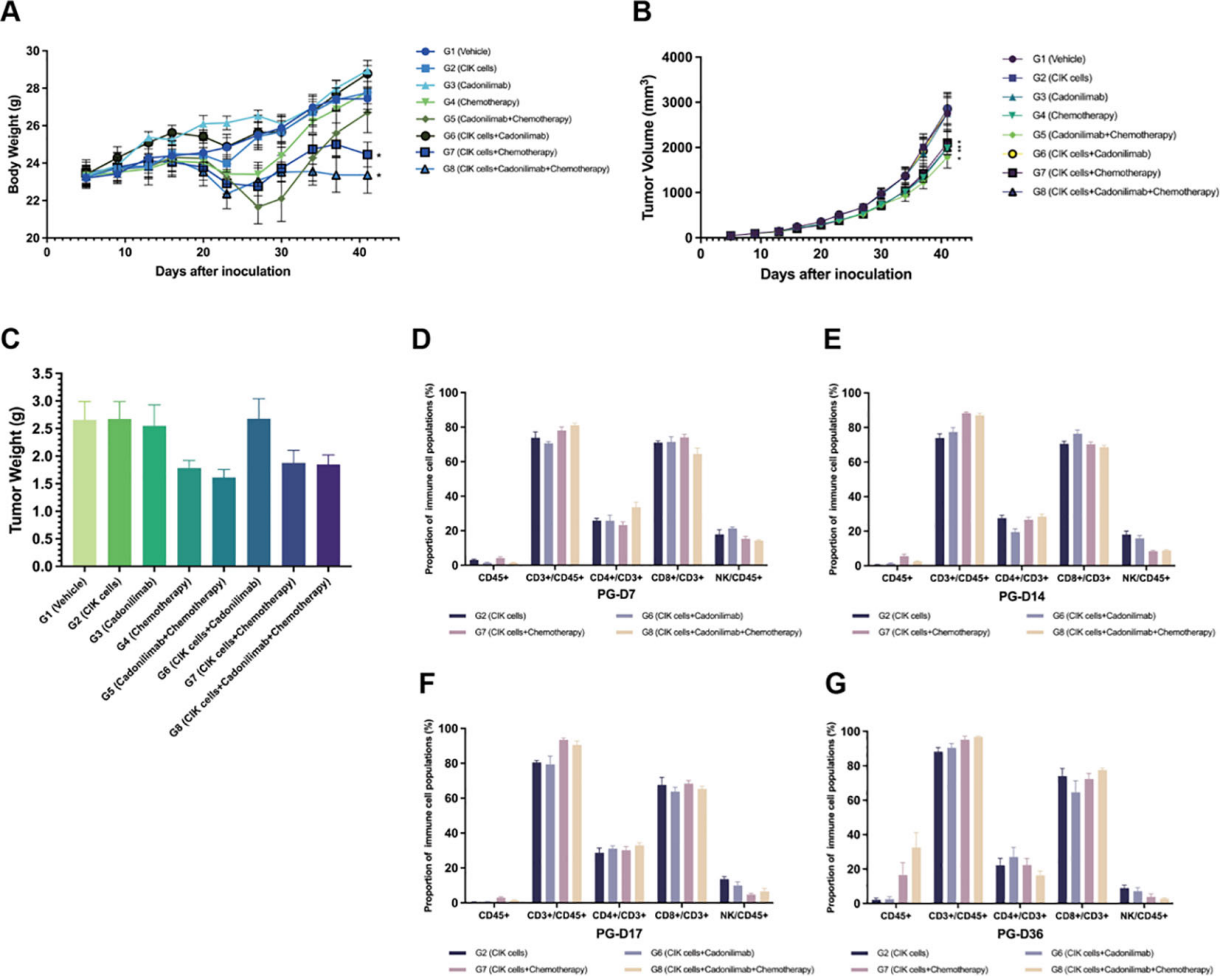
Antitumor effect of the combination of CIK cells with chemotherapy and cadonilimab. **(A)** The body weight change curve of mice after administration. **(B)** Tumor volume for each group of mice after administration. **(C)** Tumor weight of mice 36 days after administration. **(D–G)** The proportion of immune cell populations in the peripheral blood between different groups at various time points (day 7, 14, 17, and 36 after grouping). *compared with the vehicle control group, p < 0.05; mean ± SEM.

Chemotherapy combined with an ICI is known to improve the survival benefits of patients with metastatic GC. Based on the attractive antitumor effect of combining CIK cells and chemotherapy, we concluded that the addition of an ICI to CIK cells and a chemotherapy regimen might optimize cancer treatment and achieve enhanced antitumor efficacy. A subcutaneous xenograft nude mouse model was established to investigate the inhibitory effects of the combination regimen of CIK cells, chemotherapy, and cadonilimab (a PD-1/CTLA-4 bispecific antibody) on tumor growth and metastasis *in vivo*. Following administration of the regimen components, mice in each group showed normal autonomic activities, behavior, and feeding, indicating that this novel regimen was safe. The BW of tumor-bearing mice was measured to assess the toxicity of the regimen. At the end of the experiment (36 d), compared to 0 d, the average BW of the G7 group (CIK cells + chemotherapy) and G8 group (CIK cells + cadonilimab + chemotherapy) was significantly different from that of the G1 group (vehicle control) (*p* < 0.05) (**Figure 5A**). The reduced BW may be associated with GVHD.

#### 3.5.2 Changes in tumor volume and TGI

At the end of the experiment, the average tumor volume of the G1 group was 2,753 ± 351 mm^3^ and those of the G2 to G8 groups were 2,857 ± 331, 2,753 ± 389, 1,986 ± 155, 1,784 ± 240, 2,857 ± 366, 2,097 ± 250, and 1,996 ± 169 mm^3^, respectively. The average tumor volume of the G4 (chemotherapy), G5 (cadonilimab + chemotherapy), G7 (CIK cells + chemotherapy), and G8 (CIK cells + cadonilimab + chemotherapy) groups was significantly smaller than that of the G1 group (*p* < 0.05) (**Table 4**). The TGI_TV_ rates of the G2 to G8 groups were -3%, -1%, 28%, 34%, -6%, 21%, and 24%, respectively. Compared to the G2 and G3 groups, the G5, G7, and G8 groups showed significantly better tumor inhibitory effects (*p* < 0.05). However, the TGI_TV_ rates showed no significant difference between the chemotherapy-based combination treatment groups (G5, G7, and G8) and the chemotherapy-alone group (G4). Furthermore, the TGITV rates showed no significant difference between the CIK + chemotherapy + cadonilimab group (G8) and CIK + chemotherapy group (G7) (*p* = 0.77). Tumor volume changes for each group of mice are shown in **Figure 5B**.

**Table 4:** Tumor volume and tumor growth inhibition rate.

Group	Tumor volume (mm^3^) ^a^		TGI_TV_ (%)	*p* ^b^	*p* ^c^	*p* ^d^	*p* ^e^	*p* ^f^	*p* ^g^	*p* ^h^
	Before administration (PG-D0)	After administration (PG-D36)								
Vehicle	45 ± 2	2753 ± 351	–	–	–	–	–	–	–	–
CIK cells	45 ± 2	2857 ± 331	-3%	0.892	–	–	–	–	–	–
Cadonilimab	45 ± 2	2753 ± 389	-1%	0.7758	0.6798	–	–	–	–	–
Chemotherapy	45 ± 2	1986 ± 155	28%	< 0.0001^***^	< 0.0001^***^	0.0006^***^	–	–	–	–
Cadonilimab + chemotherapy	44 ± 3	1784 ± 240	34%	< 0.0001^***^	< 0.0001^***^	< 0.0001^***^	0.4792	–	–	–
CIK cells + cadonilimab	44 ± 2	2857 ± 366	-6%	0.9965	0.8955	0.7726	< 0.0001^***^	< 0.0001^***^	–	–
CIK cells + chemotherapy	44 ± 2	2097 ± 250	21%	0.0002^***^	0.0001^***^	0.0011^**^	0.8279	0.3615	0.0002^***^	–
CIK cells + cadonilimab + chemotherapy	45 ± 3	1996 ± 169	24%	< 0.0001^***^	< 0.0001^***^	0.0004^***^	0.9402	0.5241	< 0.0001^***^	0.77

^a^mean ± SEM; ^b^compared with the vehicle control group; ^c^compared with CIK cells group; ^d^compared with cadonilimab group; ^e^compared with chemotherapy group; ^f^compared with cadonilimab+chemotherapy group; ^g^compared with CIK cells+cadonilimab group; ^h^compared with CIK cells+chenotherapy group. N, number of animals; TGI_TV,_ tumor growth inhibition rate based on tumor volume; ^**^ *p* < 0.01; ^***^ *p* < 0.001.

#### 3.5.3 TW inhibition rate

After the final observation on 36 d, mice were sacrificed, and their tumors were dissected and weighed. The average TW and TGI_TW_ rates of each group were calculated and compared (**Figure 5C**, **Table 5**). The TGI_TW_ rates of the G2 to G8 groups were -1%, 4%, 33%, 39%, -1%, 29%, and 30%, respectively. Based on TW, the antitumor effects were significantly better in the G4, G5, G7, and G8 groups than in the control group (*p* < 0.05). Moreover, compared to the G2 and G3 groups, the G5, G7, and G8 groups showed significantly better tumor inhibitory effects (*p* < 0.05). However, the TGI_TV_ rates were not significantly different between the chemotherapy-based combination treatment groups (G5, G7, and G8) and the chemotherapy-alone group (G4).

**Table 5:** Tumor weight and tumor growth inhibition rate.

Group	Tumor weight (g)^a^	TGI_TW_ (%)	*p* ^b^	*p* ^c^	*p* ^d^	*p ^e^*	*p* ^f^	*p* ^g^	*p ^h^*
Vehicle	2.654 ± 0.334	–	–	–	–	–	–	–	–
CIK cells	2.673 ± 0.315	-1%	0.9593	–	–	–	–	–	–
Cadonilimab	2.548 ± 0.380	4%	0.7959	0.7591	–	–	–	–	–
Chemotherapy	1.782 ± 0.140	33%	0.0292^*^	0.0260^*^	0.0674	–	–	–	–
Cadonilimab + chemotherapy	1.610 ± 0.148	39%	0.0149^*^	0.0133^*^	0.0353^*^	0.6736	–	–	–
CIK cells + cadonilimab	2.680 ± 0.360	-1%	0.9443	0.985	0.7457	0.0250^*^	0.0127^*^	–	–
CIK cells + chemotherapy	1.875 ± 0.231	29%	0.0495^*^	0.0444^*^	0.1056	0.8094	0.5176	0.0427^*^	–
CIK cells + cadonilimab + chemotherapy	1.847 ± 0.174	30%	0.0424^*^	0.0379^*^	0.0925	0.8664	0.5626	0.0364^*^	0.9418

^a^mean ± SEM; ^b^compared with vehicle control group; ^c^compared with CIK cells group; ^d^compared with cadonilimab group; ^e^compared with the chemotherapy group; ^f^compared with cadonilimab+chemotherapy group; ^g^compared with CIK cells+cadonilimab group;**^h.^** compared with CIK cells+chenotherapy group. N, number of animals; TGI_TW,_ tumor growth inhibition rate based on tumor weight; ^*^ *p* < 0.05.

#### 3.5.4 Proportion of immune cells detected in peripheral blood samples

The proportion of immune cells in peripheral blood samples was detected by FACS. The hCD45^+^ cells showed a low proportion in the G2, G6, G7, and G8 groups at all time points. At the end of the experiment (36 d), the proportion of hCD45^+^ cells in the peripheral blood of mice in the G7 and G8 groups increased significantly (16.54% and 32.49%, respectively) as compared to that in the G6 group. Moreover, the percentage of hCD45^+^ cells was higher in the G8 group than in the G7 group, although the difference was not statistically significant (*p* > 0.05). These results suggested that ICIs combined with chemotherapy might activate CIK cells.

The majority of hCD45^+^ cells in peripheral blood samples were CD3^+^ lymphocytes. The percentage of CD3^+^ lymphocytes showed a tendency to increase after administration. In the G7 and G8 groups, in particular, the percentage of CD3^+^ cells was higher than that in the G2 and G6 groups at each time point. Among the CD3^+^ cell subsets, the percentage of CD8^+^ T cells was higher than that of CD4^+^ T cells in the four groups. NK cells formed a small subset of immune cells with a declining trend over time. The proportion of immune cells detected in peripheral blood samples between different groups is shown in **Figures 5D–G**.

## 4 Discussion

CIK cells have been extensively evaluated in preclinical studies and have shown promising antitumor effects against various tumors, including GC (27, 28). Our *in vitro* cytotoxicity assay also indicated that CIK cells showed cytotoxicity against several GC cell lines. The acute toxicity study revealed that CIK cells at moderate and low doses did not show any critical cytotoxicity, while a high dose of CIK cells caused xGVHD-related symptoms and histopathological changes. In clinical practice, autologous CIK cells do not induce GVHD, as confirmed by several clinical trials (25). Overall, CIK cells are good candidates for cellular immunotherapy in patients with GC. However, CIK cells exhibit only modest therapeutic efficacy when used as a single agent. This might be because the TME is immunosuppressive, which prevents the infiltration of CIK cells (29, 30).

Based on previous studies, conventional chemotherapy might mediate tumor cell sensitivity to ACT through multiple immune-based mechanisms (31). First, chemotherapy might play a critical role in rebounding immune cell pools after lymphodepletion, contributing to the prolonged persistence and enhanced efficacy of CIK cell therapy. Second, chemotherapy alters the TME to activate T cells for cancer cell apoptosis through low-affinity epitopes. Third, several chemotherapeutic drugs can increase CTL-induced production of IFN-γ in the TME and promote cell-mediated antitumor activity by eliminating myeloid-derived suppressor cells (MDSCs) (32). Fourth, chemotherapy can deplete immunosuppressive regulatory T cells and MDSCs (33). Fifth, the phagocytosis of cell debris enhances Toll-like receptor-dependent signaling, which directly stimulates antigen-presenting cells, thereby eliciting potent anticancer immune responses. Several preclinical studies have confirmed that chemotherapy can induce autophagy in NSCLC cells (34, 35). Therefore, we hypothesized that CIK cells can synergize with chemotherapeutic drugs to provide a robust anticancer immune response.

Combining S-1 with oxaliplatin (designated as SOX) is one of the most commonly used first-line chemotherapies for GC patients. The present research showed that the CIK cells + SOX and SOX-alone groups exhibited significantly higher tumor growth inhibition rates and well-tolerable toxicity as compared to the control group. Additionally, the antitumor effect of the CIK cells + SOX group was stronger, although not statistically significant, than that of the SOX-alone group. Thus, it can be preliminarily inferred that combining adoptive CIK cells and chemotherapy had a synergistic tumoricidal effect. In the CIK cells + SOX group, infiltrated T cells were detected at the margin of tumor tissues; this finding demonstrated that CIK cells possessed the capacity of homing to tumors and then activated the immune system to suppress tumor growth. The elevated IFN-γ secretion level and reduced levels of TNF-α and IL-6 detected in tumor tissues suggested that activated CIK cells infiltrated into tumors.

Based on a research study by Yost et al., the T cell response to ICIs relies on reinvigorating the recruitment of novel T cells rather than using preexisting tumor-infiltrating lymphocytes (36). The authors performed paired single-cell RNA and T-cell receptor sequencing of cells from site-matched tumors of patients with basal cell carcinoma or squamous cell carcinoma before and after anti-PD-1 therapy. The results showed that T cells responding to ICIs originated from a distinct repertoire of T cell clones that recently infiltrated the tumor, while pre-existing tumor-specific T cells exhibited limited reinvigoration capacity. Therefore, compared to pre-existing tumor-infiltrating lymphocytes, CIK cells as exotic T cells may respond strongly to ICI therapy and subsequently exert an overt antitumor effect. Another preclinical study showed that the combination of anti-PD-1 and anti-CTLA-4 antibodies with CIK cells exerts synergistic antitumor effects on renal cancer cells (37). Anti-PD-1 and anti-CTLA-4 antibodies induced the proliferation of CIK cells and upregulated the secretion of the immunostimulatory cytokine IFN-γ by these cells. Therefore, CIK cells combined with nivolumab and ipilimumab could be considered a promising strategy for adoptive immunotherapy.

Cadonilimab is an anti-PD-1/CTLA-4 bispecific antibody. The phase III randomized clinical trial COMPASSION-15 showed that cadonilimab plus chemotherapy significantly improved the median OS and median PFS of patients with unresectable G/GEJ adenocarcinoma (6). In our study, compared to the CIK cell group and cadonilimab group, the chemotherapy-based combination groups showed significantly better tumor inhibitory effects with tolerable toxicity. However, the chemotherapy-based regimen groups showed no significant difference in tumor lysis ability. The proportion of hCD45^+^ cells in the peripheral blood of mice was significantly increased in the CIK cells + chemotherapy group and the CIK cells + cadonilimab + chemotherapy group as compared to that in the CIK cells + cadonilimab group. The percentage of hCD45+ cells in the CIK cells + cadonilimab + chemotherapy group was also higher than that of the CIK cells + chemotherapy group, although the difference was not statistically significant. These results suggested that cadonilimab combined with chemotherapy might activate CIK cells.

Because changes in BW indirectly reflect the toxicity of antitumor drugs, we assessed the safety of the different combinations by measuring changes in the BW of treated mice. The SOX and CIK cells + SOX groups in Experiment 4 as well as the CIK cells + SOX and CIK cells + cadonilimab + SOX groups in Experiment 5 showed an apparent decrease in BW as compared to the control group. All mice in these groups received chemotherapy, and the significant decrease in BW is probably due to chemotherapy-induced toxicity and tumor shrinkage. In mice receiving CIK cells, the decreased BW might also be related to GVHD, which has been demonstrated in previous studies (38, 39). However, in clinical practice, autologous CIK cells do not cause GVHD in humans.

This study had some limitations. First, this study employed an immunodeficient mouse model, utilized CIK cells without targeting property, and used MKN45 cells, which was a human-derived cell line. Thus, this model might not appropriately reflect the immune responses related to cancer treatment; consequently, the findings of the current study cannot be fully translated to human subjects. Future studies with human subjects are required to confirm our findings. Second, the tumor PD-L1 expression level was not determined in the MGC803 cell line. In recent years, several clinical trials have confirmed that the combination of ICIs and chemotherapy provides a better prognosis for GC patients with high levels of PD-L1 expression. Hence, it is essential to accurately estimate PD-L1 expression to better understand the research data. Third, the CIK + chemotherapy regimen provided only moderate improvement in clinical response, which might be related to the short duration of administration. In published clinical trials evaluating the combination of ICI plus chemotherapy, ICI was administered for a maximum period of 2 years. The 5-year follow-up survival results of the Checkmate 649 study showed that the immunotherapy-based treatment approach could provide patients with long-term benefits. A phase II study suggested that patients who received three or more cycles of CIK cells derived most benefits from treatment with CIK cells plus chemotherapy (25). In the future, randomized controlled clinical trials are required to assess whether the addition of CIK cells to chemotherapy with ICIs could enhance the clinical benefits of patients with GC.

In conclusion, the present study showed that the combination of CIK cells and chemotherapy with or without cadonilimab served as a potential therapeutic option for treating GC. These promising preclinical data promoted the entry of this novel combination regimen into human clinical trials.

## Data Availability

The raw data supporting the conclusions of this article will be made available by the authors, without undue reservation.
