# Supplementary material for: Inhibition of human gastric cancer growth by cytokine-induced killer cells plus chemotherapy with or without cadonilimab in a mouse xenograft tumor model
Source: Front Immunol. 2025 Jun 5;16:1609320. doi: 10.3389/fimmu.2025.1609320 (PMC12176880; doi:10.3389/fimmu.2025.1609320)
Supplement: Supplementary file 1 [file DataSheet1.docx]

**Supplementary Information**

**
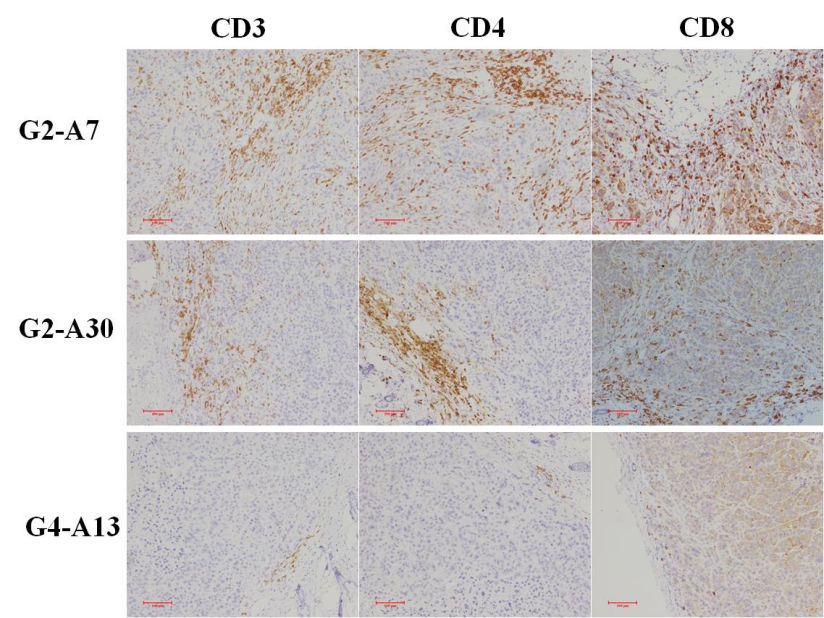
**

**Supplementary Figure 1.** Immunohistochemical analyses of T cells in tumor tissues (magnification, 🞨100). Marker-positive cells were stained brown or tan, while cell nuclei were counterstained with hematoxylin (blue staining).
